# Supplementary material for: 1H HRMAS NMR Metabolomics for the Characterization and Monitoring of Ripening in Pressed-Curd Ewe’s Milk Cheeses Produced Through Enzymatic Coagulation
Source: Foods. 2025 Jul 2;14(13):2355. doi: 10.3390/foods14132355 (PMC12248438; doi:10.3390/foods14132355)

Table S1. Manufacture characteristics of the studied varieties of Spanish sheep cheese.

		I-CLM	T-CLM	I-CL	T-CL
**Production location**		Castilla-La-Mancha (Central Spain)	Castilla-La-Mancha (Central Spain)	Castilla y León (north-western Spain)	Castilla y León (north-western Spain)
**Manufacture**		In large enterprises, according to PDO Manchego cheese	In local small enterprises according to traditional manufacture of ewe´s milk cheese	In large enterprises, according to Castellano cheese quality mark	In local small enterprises according to traditional manufacture of ewe´s milk cheese
**Commercial designation**		Manchego cheese PDO	Traditional Manchego sheep cheese	Castellano sheep cheese	Traditional Castellano sheep cheese
**Sheep breed**		Manchega	Manchega, Merina and Talaverana	Castellana and Churra	Castellana and Churra
**Milk Pre-treatment**		Pasteurised	Raw	Raw	Raw
**Starter Culture**		Homo-fermentative mesophilic LAB	Homo-fermentative mesophilic LAB	Homo-fermentative mesophilic LAB	Homo-fermentative mesophilic LAB
**Rennet**		Recombinant Chymosin	Calf commercial dried rennet	Recombinant Chymosin	Lamb commercial natural rennet
**Coagulation**	**T (°C)**	30-32	30-32	32	28-32
	**Time (min)**	45	40-45	35-40	30-45
**Cutting Curd**		Continuous automated process	Semi-continuous process	Continuous automated process	Semi-continuous process
	**Grain size (mm)**	5-10	5-10	5-8	5-10
**Mild stirring**		Continuous automated process	Discontinuous process	Continuous automated process	Discontinuous process
**Pre-drainage of whey**		Continuous automated process (mild stirred and pumped whey)	Manual (whey drainage by soft pressing)	Semi-continuous processes (mild stirred and pumped whey)	Manual (whey drainage by soft pressing)
**Scalding**	**T (°C)**	36-40	34-38	35-38	35-40
	**Time (min)**	30-35	30-40	40	30-40
**Pre-pressing**		Continuous automated process (previous pressing depending on mold size)	Manual (in curdling vats)	Continuous automated process (in curdling vats)	Manual (in curdling vats)
	**T (°C)**	30-32	30-32	30-32	30-32
	**Time (min)**	35	30	30	30
**Moulding**		Automatic	Manual	Manual	Manual
**Pressing**		Through pneumatic press	Through pneumatic press	Through pneumatic press	Through pneumatic press
	**Time (h)**	4	10-12	10-12	10
**Unmoulding**		Automated	Manual	Automated	Manual
**Salting**		By immersion in sodium chloride (20 g /100 mL)	By immersion in sodium chloride (23 g /100 ml)	By immersion in sodium chloride (20-22 g/100 mL)	By immersion in sodium chloride (20-21 g/100 mL)
	**T (°C)**	10	10-15	10	10-15
	**Time (min)**	20-24	20-24	24-30	28-32
**Ripening**		In chambers with automated control of temperature and RH	In chambers with automated control of temperature and RH	In chambers with automated control of temperature and RH	In chambers under natural conditions
	**T (°C)**	8-10	10	10	10-12
	**RH (%)**	80	75-80	75-80	75-80
	**Time (days)**	60 days for pieces bigger than 1.5 kg<br>Semi-cured: 60-90 days<br>Cured: 180 days			

I-CLM = pieces produced in an industry in Castilla-La-Mancha region; T-CLM = pieces manufactured according to an artisanal/traditional procedure also in Castilla-La-Mancha; I-CL = pieces produced in a large industry in Castilla y León; T-CL = manufactured according to an artisanal/traditional procedure also in Castilla y León. PDO = protected designation of origin. LAB = lactic acid bacteria. T (°C) = temperature; RH = relative humidity (%).

Table S2. Spectral regions (buckets) considered in the principal components (PC) analysis. Middle point and width of the spectral region, implied metabolites, and coefficient of each factor in the first two principal components (PC1 and PC2) are shown.

***Bucket* ppm**	***Bucket* width (ppm)**	**PC1**	**PC2**	**Metabolites**
8.59	0.1259	0.0968	-0.1261	Histidine
8.45	0.0908	0.1170	-0.0024	Formic acid
7.43	0.0657	0.1507	0.0312	Phenylalanine
7.38	0.0756	0.1449	-0.0075	Phenylalanine / Histidine
7.32	0.0081	0.1473	-0.0250	Phenylalanine
7.27	0.0594	0.1011	-0.0191	
7.21	0.0689	0.1461	0.0208	Tryptophan
7.12	0.1166	0.0870	-0.0475	Tyrosine
6.91	0.0829	0.1484	-0.0376	
6.82	0.1056	0.1022	-0.1001	Tyrosine
6.27	0.1463	0.0642	0.1141	CLA[a]
5.90	0.1291	0.0762	0.0878	CLA
5.76	0.1377	0.0717	0.0090	Caproleic acid
5.59	0.1330	0.0872	0.0901	CLA
5.40	0.0915	0.0437	0.1831	Trans-unsaturated fatty acid
5.31	0.0916	0.0025	-0.1465	Cis-unsaturated fatty acid
5.21	0.1150	-0.0387	-0.1318	α-Glucose / β-Galactose / CLA / Glycerol sn-1,2
5.04	0.0845	0.0246	-0.0845	Glycerol sn-1,2
4.98	0.0368	0.0258	-0.0626	Caproleic acid
4.95	0.0383	0.0097	-0.0333	β-Glucose
4.89	0.0727	-0.0117	0.0263	Caproleic acid
4.58	0.0720	0.0463	-0.1481	β-Galactose
4.43	0.1423	0.1315	-0.0895	
4.30	0.1174	0.0107	-0.1561	Glycerol sn-1,3
4.15	0.0626	0.1076	0.0618	Lactic acid
4.08	0.0978	-0.0096	-0.2034	Glycerol sn-1,3 / Choline / Asparagine
3.99	0.0899	0.1255	-0.1267	Phenylalanine / α- and β-Galactose / Tyrosine
3.89	0.1205	0.0302	-0.2017	Aspartic acid / Methionine / α-Glucose / α-Galactose
3.77	0.1228	0.0633	-0.1661	Alanine / Glutamine / Glutamic acid / Lysine
3.68	0.0625	0.0242	-0.1909	Arginine / Leucine/ α-Glucose / Isoleucine / Glycerol sn-1,3 / Ethanol
3.64	0.0274	0.0599	-0.1767	β-Glucose / β-Galactose / Glycerol sn-1,2
3.61	0.0406	0.1411	-0.0938	Valine
3.58	0.0226	0.1213	-0.1192	Threonine
3.56	0.0258	0.1224	-0.1069	Glycine / α-Glucose
3.54	0.0211	0.0707	-0.1690	Choline
3.49	0.0736	0.0033	-0.1711	β-Galactose
3.42	0.0837	0.1463	-0.0103	Proline
3.35	0.0626	0.1450	0.0042	Proline
3.31	0.0297	0.1380	-0.0393	
3.25	0.0852	0.1441	-0.0495	β-Galactose / Phenylalanine / Arginine
3.19	0.0438	0.1254	-0.0742	Choline / Tyrosine
3.15	0.0470	0.1567	-0.0160	Phenylalanine
3.11	0.0399	0.1533	-0.0171	
3.07	0.0438	0.1555	0.0006	Tyrosine
3.00	0.0923	0.1509	-0.0340	Lysine / γ-Aminobutyric acid
2.94	0.0422	0.1466	-0.0244	
2.91	0.0258	0.1319	-0.0390	Asparagine
2.88	0.0399	0.1542	0.0555	Asparagine

2.85	0.0399	0.1344	0.1228	Linoleic acid
2.81	0.0422	0.0747	0.1689	
2.78	0.0470	0.0135	-0.0607	Linolenic acid
2.74	0.0195	0.0122	-0.1837	
2.71	0.0532	0.0942	-0.0987	Citric acid
2.67	0.0211	0.1543	-0.0153	
2.64	0.0524	0.1561	-0.0199	Aspartic acid
2.60	0.0446	0.1372	-0.0625	Methionine
2.54	0.0806	0.1075	-0.0853	Citric acid
2.48	0.0540	0.1515	0.0316	
2.44	0.0469	0.1546	0.0376	Glutamine
2.38	0.0736	0.1538	0.0181	Glutamic acid
2.27	0.1517	-0.0989	-0.1755	γ-Aminobutyric acid / Valine / Fatty acids
2.14	0.0837	0.1458	0.0725	Methionine / Glutamine / Glutamic acid
2.05	0.1064	0.0460	0.0178	Unsaturated fatty acids (cis and trans)
1.98	0.0383	-0.0046	0.0114	Isoleucine
1.95	0.0235	0.0238	-0.1526	Acetic acid
1.90	0.0915	0.1410	-0.0180	γ-Aminobutyric acid / Lysine / Arginine
1.82	0.0892	0.1408	-0.0455	
1.76	0.0391	0.1562	0.0176	
1.70	0.0728	0.1556	0.0041	Leucine / Lysine / Arginine
1.60	0.1502	-0.1132	-0.1528	Fatty acids
1.49	0.0884	0.1460	0.0208	Alanine / Isoleucine / Lysine
1.35	0.0767	0.0297	0.2232	Lactic acid / Threonine
1.41	0.0634	0.0789	0.1805	
1.28	0.0822	-0.0884	-0.1913	Fatty acids / Isoleucine
1.17	0.1307	-0.0134	-0.1519	Ethanol
1.05	0.0485	0.1472	0.0298	Valine
1.02	0.0297	0.1489	0.0003	Isoleucine
0.99	0.0282	0.1453	0.0874	
0.97	0.0289	0.1224	0.1505	Leucine
0.94	0.0454	0.0558	0.2118	Isoleucine / Butyric acid
0.87	0.1150	-0.0899	-0.1806	Fatty acids
0.78	0.0822	-0.0035	-0.1117	
0.72	0.0509	-0.0098	-0.1045	Cholesterol

[a]CLA = conjugated linoleic acid.

Table S3. Signals assigned in the ^{1}H HRMAS NMR spectra of cheeses (I-CL, T-CL, I-CLM and T-CLM) at different ripening times.

δ (ppm)	Compound	Group (Multiplicity)	Additional information
0.72	Cholesterol	CH_3 (s)	Scano et al. (2011)
0.91	Fatty acids	CH_3 (t)	COSY (1.30)
0.93	Butyric acid	CH_3 (t)	COSY (1.58); TOCSY (2.21)
0.94	Isoleucine	δ–CH_3	COSY (1.27;148)
0.97	Leucine	δ–CH_3/δ'–CH_3 (d)	COSY (1.72); TOCSY (3.74)
0.98	Fatty acids (ω–3)	CH=CH–CH_2 –CH_3 (t)	COSY (2.08); TOCSY (2.79; 5.35)
0.99	Valine	γ–CH_3 (d)	COSY (2.29); TOCSY (3.61)
1.02	Isoleucine	γ–CH_3 (d)	COSY (1.98); TOCSY (3.68)
1.05	Valine	γ'–CH_3 (d)	COSY (2.29); TOCSY (0.99)
1.19	Ethanol	CH_3 (t)	COSY (3.66)
1.27	Isoleucine	γ–CH_2 (m)	COSY (1.02)
1.30	Fatty acids	$(CH_2)_n$ (s)	COSY (1.60; 2.04); TOCSY (2.27;5.35)
1.33	Threonine	γ–CH_3	COSY (4.26); TOCSY (3.58)
1.34	Lactic acid	β–CH_3 (d)	COSY (4.13)
1.47	Lysine	γ–CH_2 (m)	COSY (1.71; 1.91); TOCSY (3.02)
1.48	Isoleucine	γ–CH_2	COSY (1.02)
1.48	Alanine	β–CH_3 (d)	COSY (3.78)
1.60	Fatty acids	CH_2 –CH_2–CH_2 –COOR	COSY (1.30; 2.27)
1.69	Arginine	γ–CH_2 (m)	COSY (1.90; 3.25)
1.71	Lysine	δ–CH_2	COSY (1.47; 3.02); TOCSY (1.91)
1.72	Leucine	γ–CH	COSY (0.97)
1.72	Leucine	β–CH_2	COSY (3.74)
1.90	Arginine	β–CH_2 (m)	COSY (1.69; 3.76)
1.91	Lysine	β–CH_2	COSY (1.47; 3.77); TOCSY (1.71)
1.91	γ–Aminobutyric acid	β–CH_2 (q)	COSY (2.31; 3.01)
1.93	Acetic acid	CH_3 (s)	Castejón et al. (2010)
1.98	Isoleucine	β–CH	COSY (1.02)
2.04	Unsaturated fatty acids	CH_2(trans)–H=CH– (m)	COSY (1.30; 5.35); TOCSY (2.80)
2.08	Unsaturated fatty acids	CH_2(cis)–CH=CH– (m)	COSY (0.98; 3.35); TOCSY (2.83)
2.13	Glutamic acid	β–CH_2	COSY (2.36; 3.77)
2.13	Methionine	S–CH_3	Castejón et al. (2010)

2.14	Glutamine	β,β'–CH_2	COSY (2.44; 3.78)
2.18	Methionine	β–CH_2	COSY (2.64; 3.88)
2.27	Fatty acids	CH_2– CH_2– COOR	COSY (1.60); TOCSY (1.30)
2.29	Valine	β–CH (m)	COSY (0.99; 1.05; 3.61)
2.31	γ–Aminobutyric acid	α–CH_2 (m)	COSY (1.91)
2.36	Glutamic acid	β–CH_2	COSY (2.13); TOCSY (3.77)
2.44	Glutamine	β–CH_2 (m)	COSY (2.14; 3.78)
2.54	Citric acid	CH_2	COSY (2.71)
2.63	Methionine	γ –CH_2	COSY (2.18); TOCSY (3.85)
2.70	Aspartic acid	β–CH_2	COSY (3.90)
2.71	Citric acid	CH_2	COSY (2.54)
2.79	Linoleic acid	CH_2–CH=CH–CH_2–CH=CH–CH_2 (m)	COSY (3.35); TOCSY (0.98; 1.30; 2.08)
2.83	Linoleic acid	CH_2=CH–CH–CH_2=CH–CH=CH_2 (m)	COSY (3.35); TOCSY (2.04)
2.86	Asparagine	β–CH_2 (dd)	COSY (2.90)
2.90	Asparagine	β’-CH_2 (dd)	COSY (2.86)
3.01	γ–Aminobutyric acid	γ–CH_2 (m)	COSY (1.91)
3.02	Lysine	ε–CH_2 (m)	COSY (1.71); TOCSY (1.47; 1.91; 3.77)
3.06	Tyrosine	β–CH_2	COSY (3.94)
3.13	Phenylalanine	β–CH_2	COSY (3.99)
3.21	Tyrosine	β–CH_2	COSY (3.94)
3.21	Choline	N–CH_3 (s)	Piras et al. (2013)
3.26	Arginine	δ–CH2 (t)	COSY (1.69); TOCSY (1.90; 3.76)
3.27	Phenylalanine	β–CH_2	COSY (3.99)
3.27	β–Glucose	CH–2 (m)	COSY (4.65; 3.65)
3.37	Proline	δ–CH (t)	Piras et al. (2013)
3.42	Proline	δ´–CH (t)	Piras et al. (2013)
3.48	β–Galactose	CH–2 (m)	COSY (4.57); TOCSY (3.92)
3.54	Choline	β–CH_2	COSY (4.07)
3.55	α–Glucose	CH–2 (m)	COSY (5.22)
3.55	Glycine	α –CH_2 (s)	Castejón et al. (2010)
3.58	Threonine	α–CH (d)	COSY (4.26); TOCSY (1.33)
3.61	Valine	α–CH (d)	COSY (2.29); TOCSY (0.99; 1.05)

3.62	Glycerol sn-1,2 diacylglycerol	HO–CH_2–CH– (d)	Scano et al. (2011)
3.63	β–Galactose	CH–3 (m)	TOCSY (4.57)
3.65	β–Glucose	CH–3 (m)	COSY (3.27); TOCSY (4.65)
3.66	Ethanol	CH_2 (q)	COSY (1.19)
3.69	Glycerol sn-1,3 diacylglycerol	–CH_2–OOC– (m)	Scano et al. (2011)
3.68	Isoleucine	α–CH	COSY (1.98); TOCSY (1.02)
3.70	α–Glucose	CH–3 (m)	TOCSY (5.22)
3.74	Leucine	α–CH (m)	COSY (1.72); TOCSY (0.97)
3.75	Arginine	α–CH_2 (t)	COSY (1.90); TOCSY (1.69; 3.25)
3.77	Lysine	α–CH (t)	COSY (1.91); TOCSY (3.02)
3.77	Glutamic acid	α–CH (m)	COSY (2.11); TOCSY (2.36)
3.78	Glutamine	α–CH (m)	COSY (2.14); TOCSY (2.44)
3.78	Alanine	α–CH	COSY (1.48)
3.82	α–Galactose	CH–2 (m)	COSY (5.25)
3.84	α–Galactose	CH–3 (m)	TOCSY (5.25)
3.85	Methionine	α–CH	COSY (2.18)
3.87	α–Glucose	CH–4 (m)	TOCSY (5.22)
3.90	Aspartic acid	α–CH	COSY (2.70)
3.92	β–Galactose	CH-4 (m)	TOCSY (3.48; 4.57)
3.94	Tyrosine	α–CH	COSY (3.06; 3.21)
3.98	α–Galactose	CH–4 (m)	TOCSY (5.25)
3.99	Phenylalanine	α–CH (dd)	COSY (3.13; 3.27)
4.01	Asparagine	α–CH (dd)	Piras et al. (2013)
4.07	Choline	α–CH_2	COSY (3.54)
4.12	Glycerol sn-1,3 triacylglycerol	CH_2–OOC (dd)	COSY (4.30; 5.26)
4.13	Lactic acid	α–CH (q)	COSY (1.34)
4.26	Threonine	α–CH (m)	COSY (3.58)
4.30	Glycerol sn 1,3 triacylglycerol	CH_2–OOC–(dd)	COSY (4.12; 5.26)
4.57	β–Galactose	CH–1 (d)	COSY (3.48); TOCSY (3.65; 3.92)
4.65	β–Glucose	CH-1 (d)	COSY (3.27); TOCSY (3.65)
4.90	Caproleic acid	=CH- (dd)	COSY (5.77)
4.97	Caproleic acid	=CH- (dd)	COSY (5.77)
5.08	Glycerol sn 1,2 triacylglycerol	–CH–OOC– (m)	COSY (4.30)
5.22	α–Glucose	CH-1 (d)	COSY (3.55); TOCSY (3.70; 3.87)

5.26	Glycerol sn 1,2 triacylglycerol	–CH–OOC– (m)	COSY (4.12; 4.30)
5.27	Conjugated linoleic acid	=CH-	COSY (5.93); TOCSY (5.27; 6.26)
5.25	α–Galactose	CH–1 (d)	COSY (3.82); TOCSY (3.84; 3.98)
5.35	Unsaturated fatty acid	–CH (cis)=CH– (m)	COSY (2.04; 2.79; 2.83); TOCSY (0.98; 1.30; 2.35)
5.37	Unsaturated fatty acid	–CH (trans)=CH– (m)	Scano et al. (2011)
5.65	Conjugated linoleic acid	–CH= (dd)	COSY (5.65; 5.93); TOCSY (5.65)
5.77	Caproleic acid	$–CH=CH_2$ -(m)	COSY (4.90; 4.97)
5.93	Conjugated linoleic acid	–CH= (t)	COSY (5.27; 6.28); TOCSY (5.65)
6.28	Conjugated linoleic acid	–CH= (dd)	COSY (5.65; 5.93); TOCSY (5.27)
6.89	Tyrosine	CH–3,5 (d)	COSY (7.17)
7.17	Tyrosine	CH–2,6 (d)	COSY (6.89)
7.19	Tryptophan	CH–6	TOCSY (7.53)
7.32	Phenylalanine	CH–2,6 (m)	COSY (7.42)
7.33	Histidine	CH–4 (s)	COSY (8.52)
7.39	Phenylalanine	CH–3,5 (m)	COSY (7.42)
7.42	Phenylalanine	CH–4 (m)	COSY (7.32)
7.53	Tryptophan	CH–t	TOCSY (7.19)
8.42	Formic acid	CH (s)	Castejón et al. (2010)
8.52	Histidine	CH–2 (s)	COSY (7.33)

Table S4. Principal component coefficients of the projection based on the spectral regions corresponding to amino acids from the ^{1}H HRMAS NMR spectra regarding the distribution of Manchego cheese (CLM) samples produced by industrial (I) and traditional (T) methods at different ripening times (days) (Figure 3B).

	PC1	PC2
His	0.25	-0.33
Phe	0.30	-0.02
Trp	0.28	0.27
Tyr	0.27	-0.27
Val	0.27	-0.32
Thr	0.29	-0.11
Pro	0.29	0.21
Asp	0.28	0.32
Met	0.28	-0.26
Gln	0.29	-0.12
Glu	0.30	-0.06
Ile	0.30	0.19
Leu	0.20	0.60

Asp: Aspartic acid, Pro: Proline, Ile: Isoleucine, Trp: Tryptophan, Leu: Leucine, Val: Valine, Thr: Threonine, His: Histidine, Met: Methionine, Phe: Phenylalanine, Tyr: Tyrosine, Gln: Glutamine, Glu: Glutamic acid.

Table S5. Principal component coefficients of the projection based on the spectral regions corresponding to amino acids from the ^{1}H HRMAS NMR spectra regarding the distribution of pressed-curd cheese samples made from ewe's milk using enzymatic coagulation—Manchego (CLM) and Castellano (CL)—produced by industrial (I) and traditional (T) methods at different ripening times (days) (Figure 4).

	PC1	PC2
His	0.29	-0.08
Phe	0.33	-0.04
Trp	0.32	-0.06
Tyr	0.31	0.12
Val	0.32	0.02
Thr	0.29	-0.16
Pro	0.29	-0.23
Asp	0.32	-0.03
Met	0.26	0.37
Gln	0.25	0.38
Glu	0.11	0.53
Ile	0.30	-0.23
Leu	0.09	-0.53

Asp: Aspartic acid, Pro: Proline, Ile: Isoleucine, Trp: Tryptophan, Leu: Leucine, Val: Valine, Thr: Threonine, His: Histidine, Met: Methionine, Phe: Phenylalanine, Tyr: Tyrosine, Gln: Glutamine, Glu: Glutamic acid.

Figure S1. Evolution of ^{1}H HRMAS NMR spectra during ripening of T-CLM cheese

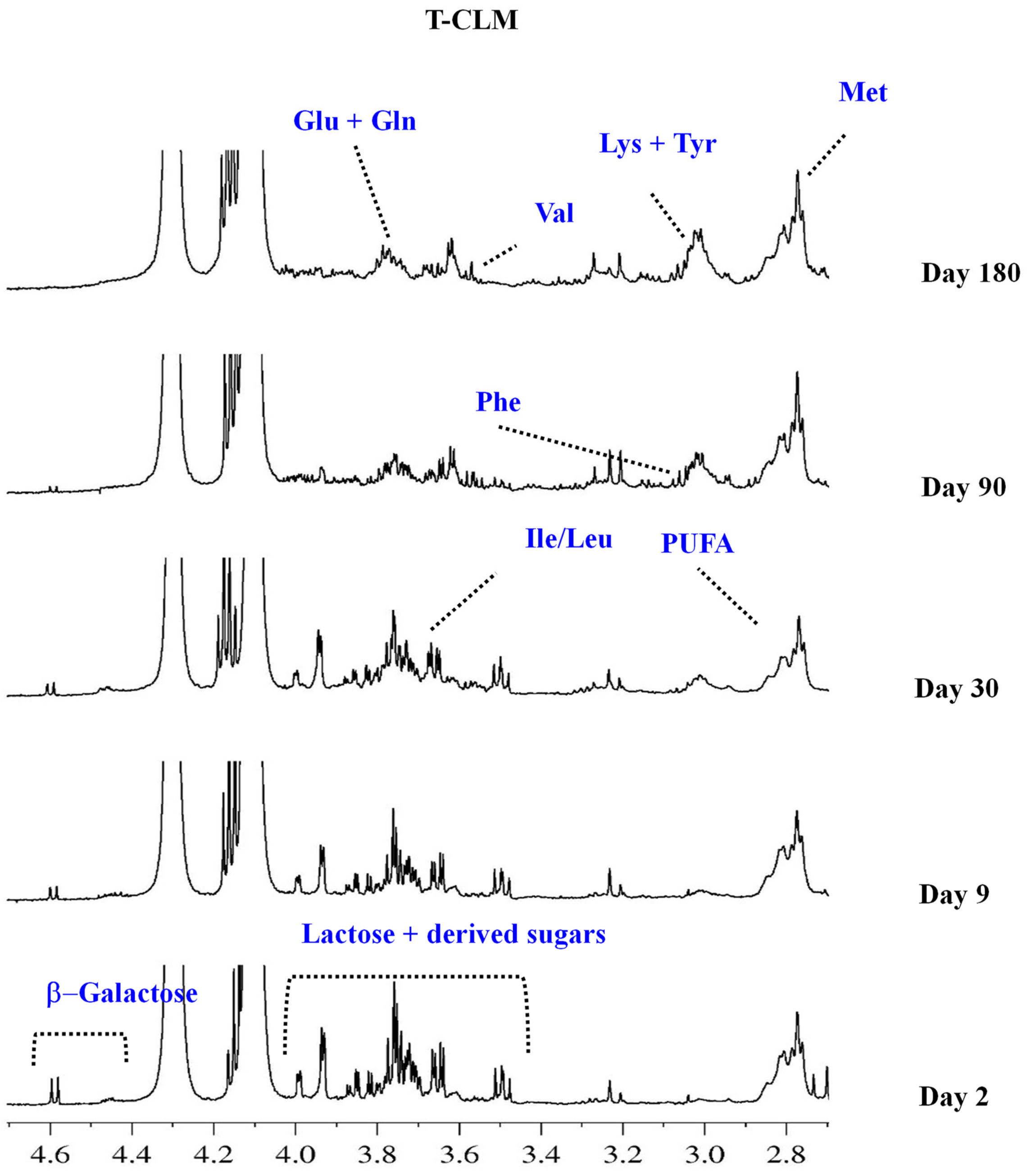

Figure S2. Distribution of the variables corresponding to the spectral regions of the ^{1}H HRMAS NMR spectra used in the cluster analysis of Manchego (CLM) and Castellano (CL) ewe's milk cheese samples, produced by industrial (I) and traditional (T) methods at different ripening times.

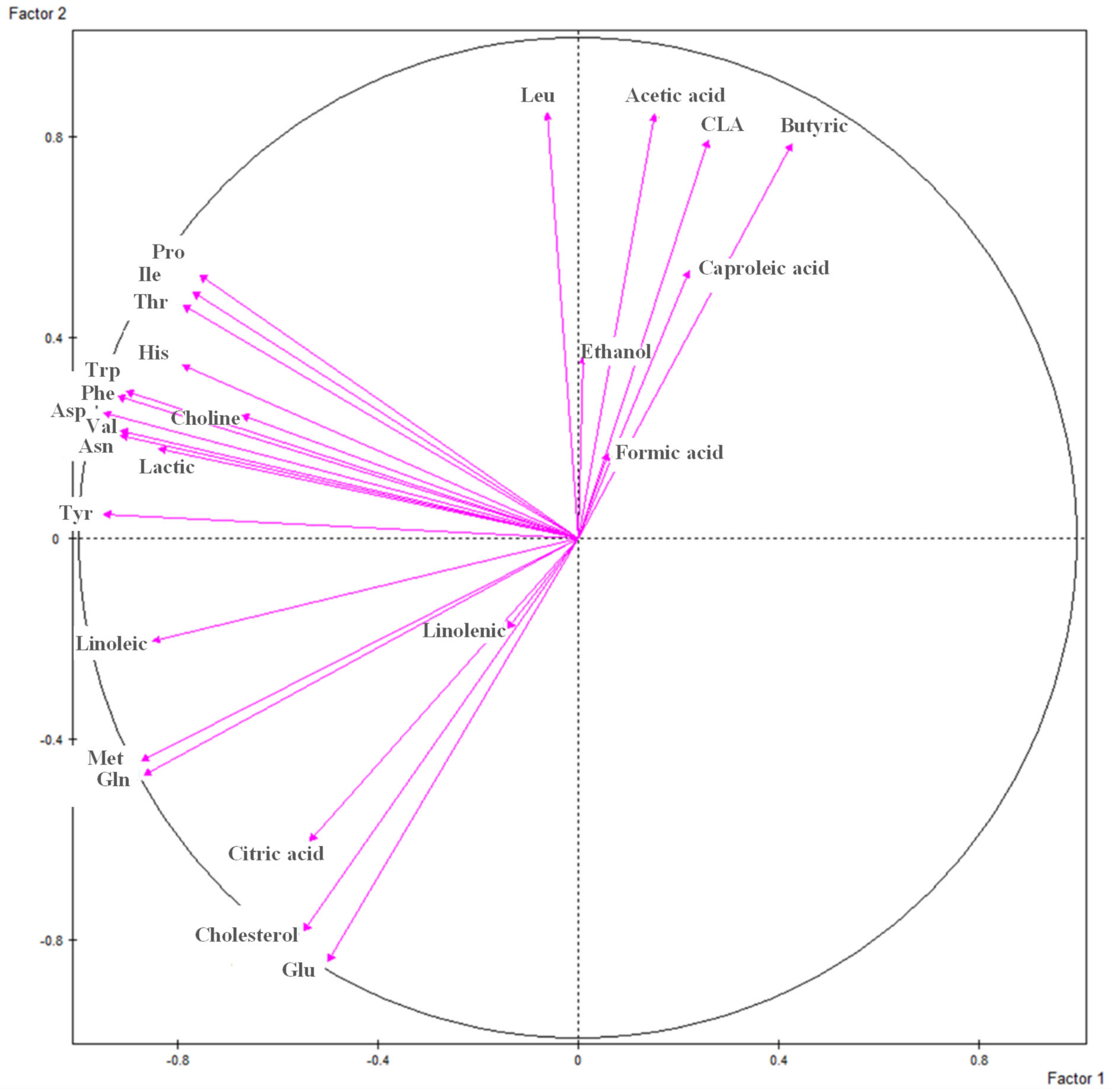

Supplement: Supplementary file 1 [file foods-14-02355-s001.zip › foods-3711300-supplementary.pdf]
